# Revealing the secrets of neuronal circuits with recombinant rabies virus technology

**DOI:** 10.3389/fncir.2013.00002

**Published:** 2013-01-24

**Authors:** Melanie Ginger, Matthias Haberl, Karl-Klaus Conzelmann, Martin K. Schwarz, Andreas Frick

**Affiliations:** ^1^Neurocentre Magendie, INSERM U862Bordeaux, France; ^2^University of BordeauxBordeaux, France; ^3^Institute of Neuroinformatics, University of ZürichZürich, Switzerland; ^4^Max-von-Pettenkofer Institute and Gene Center of the Ludwig-Maximilians University MunichMunich, Germany; ^5^Department of Molecular Neurobiology, Max Planck Institute for Medical Research HeidelbergHeidelberg, Germany

**Keywords:** connectivity, wiring diagram, neuronal tracer, synapses, monosynaptic

## Abstract

An understanding of how the brain processes information requires knowledge of the architecture of its underlying neuronal circuits, as well as insights into the relationship between architecture and physiological function. A range of sophisticated tools is needed to acquire this knowledge, and recombinant rabies virus (RABV) is becoming an increasingly important part of this essential toolbox. RABV has been recognized for years for its properties as a synapse-specific trans-neuronal tracer. A novel genetically modified variant now enables the investigation of specific monosynaptic connections. This technology, in combination with other genetic, physiological, optical, and computational tools, has enormous potential for the visualization of neuronal circuits, and for monitoring and manipulating their activity. Here we will summarize the latest developments in this fast moving field and provide a perspective for the use of this technology for the dissection of neuronal circuit structure and function in the normal and diseased brain.

## Introduction

How does the brain process information, encode perception, and generate behavior? An answer to these questions requires a mechanistic understanding of the operation of the brain's neural circuits. The operation of these neural circuits is determined by their structure, the physiological properties of the neuronal connections, and the integrative properties of the neurons. Thus, an understanding of the brain's computations is inevitably linked to knowledge of the relationship between the structure of its neuronal circuits and their function (Lichtman and Denk, 2011; Rancz et al., 2011). Rabies virus (RABV) has outstanding properties as a retrograde tracer of synaptically connected neuronal populations (Kelly and Strick, 2000; Wickersham et al., 2007a; Ugolini, 2011). Recently, the development of a glycoprotein gene-deleted (ΔG) RABV based method has enabled the tracing and functional investigation of monosynaptic connections of defined neurons (Wickersham et al., 2007b; Callaway, 2008; Marshel et al., 2010). This methodology can be readily combined with the expression of a variety of genes, optical/electrophysiology methods, and behavioral assays to drive integrative studies of neural circuits (Callaway, 2008; Arenkiel and Ehlers, 2009; Osakada et al., 2011; Wickersham and Feinberg, 2012).

In this review we present an update on recent developments in the field of RABV ΔG based trans-synaptic tracing technology. We explain the basic principles of this technology, and show how, in recent studies, it has been adapted to reveal specific aspects of neuronal circuit structure/function. We also discuss what we believe to be the major potential and shortcomings of recombinant RABV technology. Lastly, we provide an overview of how this approach complements other recent technological developments in the field of neural circuit analysis and the kind of questions that can be addressed using a combination of these approaches.

## Trans-synaptic tracers for the study of neural circuit connectivity

### Polysynaptic tracers

Conventional anterograde and retrograde tracers have significantly advanced our knowledge of connectivity between different brain areas (Cowan, 1998; Luo et al., 2008; Lichtman and Denk, 2011). These tracers reveal which brain areas are connected to one another and the location and identity of the projections neurons. However, since these tracers do not cross synapses, they are unable to establish direct synaptic connectivity between neurons.

Polysynaptic tracers, on the other hand, have the ability to spread from one neuron to another, permitting the identification of networks of synaptically connected neurons. These polysynaptic tracers can be divided into two types: non-viral and viral tracers. Non-viral polysynaptic tracers are, for the most part, limited to certain types of plant lectin and bacterial toxins (Cowan, 1998; Carter and Shieh, 2010). These tracers have a number of advantages including their safety, ease of use and ability to be genetically encoded. However, they also suffer from one or more shortcomings (reviewed in references Callaway, 2008; Luo et al., 2008; Lichtman and Denk, 2011; Wickersham and Feinberg, 2012), importantly severe dilution of signal with each synaptic step.

These limitations can be overcome by the use of certain polysynaptic viral tracers, in particular those belonging to the classes of α-herpes viruses (herpes simplex virus type 1 and pseudorabies virus) and RABV (reviewed in Callaway, 2008; Ekstrand et al., 2008). In contrast to non-viral tracers, trans-synaptic viral labeling is amplified rather than diluted due to the replicative nature of viruses. This labeling can be revealed by the expression of suitable morphological markers such as native viral proteins or reporter molecules in the case of recombinant virus strains (reviewed in Ekstrand et al., 2008). Not all of the polysynaptic viral tracers spread exclusively at synaptic sites, thereby labeling neurons that are not necessarily connected by synapses. The CVS derived strains of RABV and the Bartha strain of pseudorabies virus (PRV), however, have been shown to spread in a synapse-specific manner (reviewed in references Callaway, 2008; Ekstrand et al., 2008; Luo et al., 2008; Ugolini, 2011). Furthermore, these two viruses spread in a defined (retrograde) direction, enhancing their value for neural circuit analysis. RABV has two main advantages over PRV, namely a significantly reduced cytotoxicity and the ability to be used in primates (Callaway, 2008). The relative merits of these polysynaptic viruses for neural circuit analysis have been discussed in detail elsewhere (Callaway, 2008; Ekstrand et al., 2008; Ugolini, 2011).

Altogether, these features make RABV a very useful virus for the study of neural circuit connectivity (Ugolini, 1995; Kelly and Strick, 2000; Callaway, 2008; Ugolini, 2011). However, the fact that native RABV (like other trans-synaptic viruses) is a polysynaptic tracer causes potential ambiguity in the interpretation of how many synaptic steps have been crossed at any given time. For instance, such ambiguity would occur if RABV crosses synapses at different rates depending on their strength or simply because of differences in the RABV transport time along presynaptic axons that span several orders of magnitude in length (for discussion of synapse strength see references Ugolini, 1995, 2011; Callaway, 2008; Wickersham and Feinberg, 2012). Furthermore, it is not possible to concomitantly use this method for both circuit tracing and the manipulation of first-order connections (see below), since higher-order connections will also be affected.

### RABV as a monosynaptic tracer

#### Revealing first-order connections

An elegant solution to this problem led to a technique, which has come to be known as monosynaptic trans-synaptic tracing (Wickersham et al., 2007b) or mono-trans-synaptic tracing for short (Miyamichi et al., 2011). Mono-trans-synaptic tracing uses a pseudotyped, recombinant RABV to identify direct presynaptic inputs to a defined target cell population. These connections are “mono-synaptic” because trans-synaptic traversal is limited to one synaptic step between initial infected cells (i.e., the source cells) and their immediate presynaptic partners. The exquisite specificity of the RABV ensures that only synaptically connected neurons are labeled.

**Figure 1:**
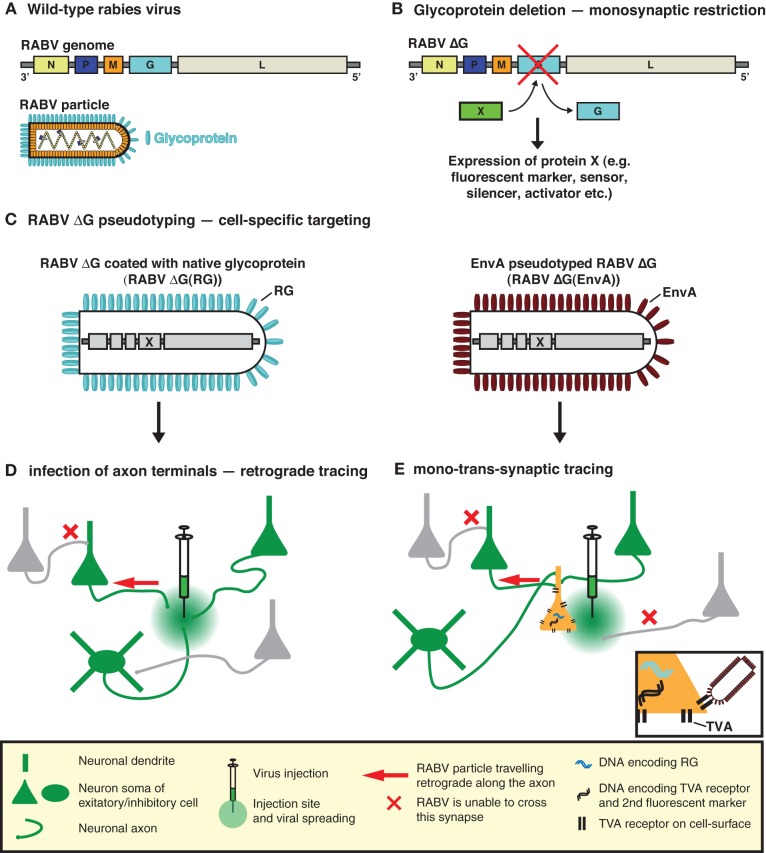
**RABV—the trans-synaptic tracing toolbox. (A)** The RABV genome encodes five proteins including the envelope glycoprotein (G). **(B)** Deletion of G (ΔG) is sufficient to prevent trans-synaptic spreading and results in a single-cycle vector. A gene of interest can then be inserted in the place of the deleted G. This gene of interest can encode a fluorescent protein such as GFP, permitting the visualization of virally traced neurons. It can also permit the expression of an almost unlimited choice of genetically encoded tools for the manipulation/visualization of neuronal circuits, e.g., biosensors, synapse markers, activators/repressors of neuronal activity. **(C)** RABV ΔG can be pseudotyped, either with its native glycoprotein or an engineered surface protein (e.g., EnvA). Pseudotyping restores the normal infection capabilities or enables cell-type selective infection, respectively. **(D)** RABV ΔG coated with its native glycoprotein is taken up by axon terminals and can be used to trace neurons in a retrograde manner. **(E)** EnvA pseudotyped RABV ΔG [RABV ΔG(EnvA)] only infects neurons expressing the TVA receptor. Trans-complementation with RG enables RABV ΔG to cross one synaptic step to infect the presynaptic partners of a defined neuron or neuronal population. The virus is then trapped in these presynaptic partner cells and cannot spread further due to the deficiency of RG in these presynaptic cells, hence limiting the strategy to a mono-trans-synaptic event. Initially infected source cells are identified by the expression of an additional fluorescent marker.

To implement this highly specific tracing system, Wickersham et al. (2007b) employed a deletion mutant RABV (RABV ΔG) (Mebatsion et al., 1996a), deficient in the expression of the RABV envelope glycoprotein (RG) (Figures 1A,B). RG is essential for the assembly of infectious virus particles during the natural life cycle of RABV (Mebatsion et al., 1996a), as well as for mediating trans-synaptic crossing of the virus (Mebatsion et al., 1996a; Etessami et al., 2000) (Figure 1B). Infectious properties can be rescued by cultivating the virus in a trans-complementing cell line (pseudotyping; Figures 1C,D). The resulting virus, however, behaves as a single-cycle tracer and is trapped in the source cells. Exogenous expression of RG in the same source cell population is sufficient to rescue the trans-synaptic capabilities of the virus—but in a highly restricted manner, which is limited to first order connections (Wickersham et al., 2007b) (Figure 1E).

In the original description of the method, pseudotyping was employed as a means of limiting the initial infection to a defined source cell population. To achieve this, Wickersham et al. (2007b) exploited the envelope glycoprotein (EnvA) of the avian sarcoma and leukosis virus, whose cognate receptor (TVA) has no homologue in mammalian cells (Bates et al., 1993; Young et al., 1993; Federspiel et al., 1994). Ectopic TVA expression is required to permit infection by RABV ΔG(EnvA) and needs to occur in the same cells expressing RG, to correctly demarcate the source cell population (Figure 1E). In order to visualize the resulting trans-synaptically labeled neurons, RABV was modified to express a fluorescent protein (eGFP) and source cells are distinguished by the addition of a second fluorescent marker (Wickersham et al., 2007b) (Figure 1B). Pseudotyping can also be achieved with a variety of viral glycoproteins provided that the glycoprotein is suitably engineered to contain the cytoplasmic domain of the native RABV glycoprotein (Mebatsion et al., 1995; Conzelmann, 1998; Choi et al., 2010; Choi and Callaway, 2011). In such cases, the source cell population is defined by the resulting tropism of the pseudotyped virus.

#### Features of RABV vectors

RABV is, in many ways, the prototypic neurotropic virus since its life cycle is entirely adapted to survival and spread in the nervous system. RABV derived vectors thus possess a number of features, which endow them with excellent properties for a viral tracer. These include efficient long-range transport in neurons, low cytotoxicity, and high-level gene expression (Mebatsion et al., 1996b; Wickersham et al., 2007a; Dietzschold et al., 2008). Wild-type RABV has a broad host species range that includes almost all terrestrial mammals, bats, and birds (Gough and Jorgenson, 1976; Nel and Markotter, 2007), and this likely extends to all RABV derived vectors making them suitable for use in a range of animal model systems. The RABV genome (Conzelmann et al., 1990) is small, modular, and readily accepts foreign genes without the issues of instability inherent to other RNA viruses (Mebatsion et al., 1996b; Conzelmann, 1998). Moreover, RABV is confined to the cytoplasm throughout its life cycle (Albertini et al., 2011). This precludes integration into the host genome and the subsequent disruption or inappropriate transcriptional activation of host genes, which is reported to occur for other vectors such as lentivirus (Sakuma et al., 2012). The highly stable and tightly bound ribonucleoprotein core (Albertini et al., 2011) also prevents recombination between the RNA genome and cellular RNAs.

Transgene expression from a RABV vector is integrally tied to viral genome expression and is thus constitutively ON. RABV transcription starts at the 3′ end (upstream of the N gene; Figure 1A) and proceeds sequentially throughout the genome. Attenuation at gene boundaries generates a gradient of transcripts with respect to gene order (reviewed in references Conzelmann et al., 1990; Albertini et al., 2011), resulting in a finely tuned ratio of gene products in the native virus (reviewed in references Conzelmann, 1998; Albertini et al., 2011). This feature can be manipulated to some extent to influence the level of expression of a transgene. In addition, “engineered” gene border transcription signals (Finke et al., 2000) or regulatory elements from other RNA viruses can also be deployed to modify transgene expression levels (Marschalek et al., 2009). RABV ΔG vectors expressing a single transgene have typically made use of the endogenous transcription signals, which normally flank the glycoprotein gene (Wickersham et al., 2007b; Osakada et al., 2011). In addition, it is possible to insert additional cistrons as long as each is flanked by suitable transcription start/stop signals (Conzelmann, 1998; Osakada et al., 2011). This principle was used to express two-independent genes from a RABV ΔG vector (Osakada et al., 2011). In this case 3.6 kb of exogenous sequence was effectively integrated into the RABV ΔG viral genome and subsequently packaged into infectious particles. Unlike other vectors, the most important factor limiting “cloning capacity” is probably not the total insert size, but instead the number of additional transcription initiation start sites introduced with the exogenous genes.

The RABV genome never encompasses a DNA form and this poses particular constraints on RABV derived vectors. Certain genetic tools such as lox P sites, tet-regulatory sequences or cell-specific promoters simply do not function with a negative-sense single-stranded RNA [(−)ssRNA] virus since a double stranded DNA binding site or substrate is required. Unfortunately, this precludes the use of a number of genetic tools that are normally employed for cell-specific or inducible expression of the viral-driven transgene (see Figure 4). The (−)ssRNA RABV genome also presents practical difficulties for the production of recombinant viral particles. Although improved methods for RABV ΔG rescue have recently been described (Wickersham et al., 2010; Osakada et al., 2011; Ghanem et al., 2012), reverse genetics approaches, required for the recovery of infectious RABV ΔG from cloned DNA, remain several orders of magnitude less efficient than for positive strand RNA or DNA viruses (Schnell et al., 1994; Ghanem et al., 2012). This being said, it is worth noting that once a new recombinant form of RABV ΔG has been recovered from DNA, it is straightforward to amplify the virus using an RG-expressing cell line (Osakada et al., 2011).

## Strategies for tracing monosynaptic connections to specific brain regions, neuron types, or individual neurons

### Targeting specific brain regions

A specific brain region can simply be targeted via stereotaxic injection of CNS competent viral vectors expressing TVA/RG, followed several days later by injection into the same region of the RABV ΔG(EnvA). For example, mono-trans-synaptic tracing was crucial for determining synaptic connections between oxytocin-expressing neurons of the hypothalamic accessory magnocellular nucleus and neurons of the central amygdala (Knobloch et al., 2012).

### Targeting a defined neuronal cell population

While the aforementioned approach maps the connectivity of multiple neuron types within the respective brain structure, it may be desirable to target a defined neuronal cell population. In the next paragraphs we will highlight the variety of approaches that have been developed to achieve neuron type specific mono-trans-synaptic tracing.

**Figure 2:**
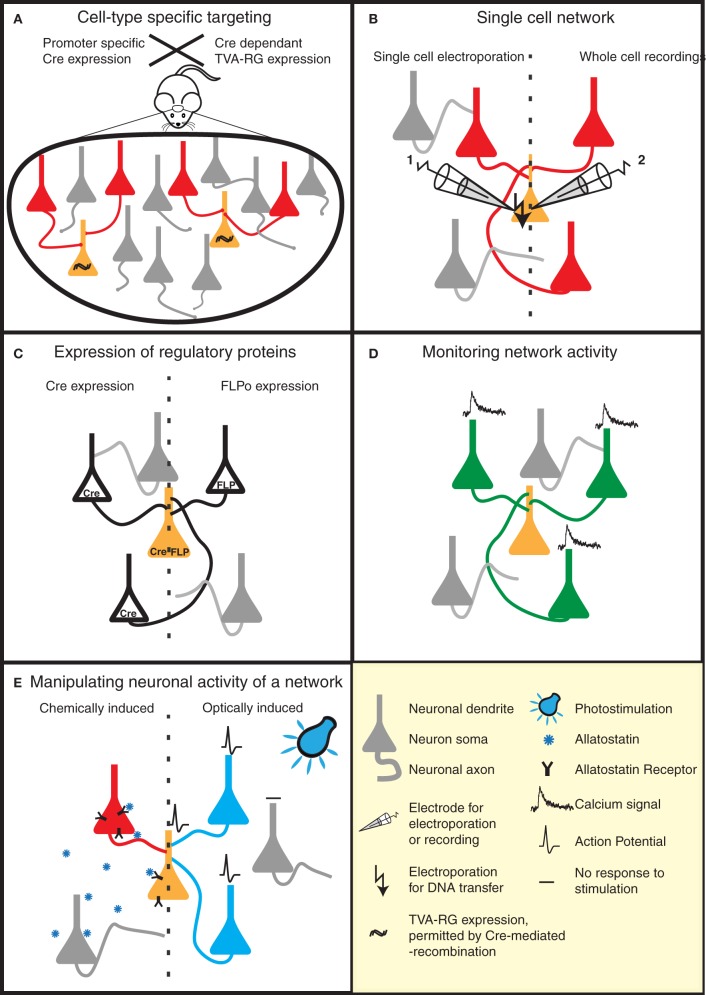
**Strategies to reveal the secrets of defined neural circuits. (A,B)** There are several strategies to target the rabies infection to individual neurons or specific cell types. **(A)** Genetic strategies such as the Cre-lox system take advantage of cell-specific promoters to restrict Cre expression to a defined cell-type. Injection of a Cre-dependent helper virus expressing RG and TVA permits RABV ΔG(EnvA) infection, and subsequent mono-trans-synaptic tracing, to be limited to a specific source cell population. **(B)** Patch pipettes can be used to infect individual neurons with the plasmids encoding RG and specific surface proteins (e.g., TVA). These neurons are subsequently infected with pseudotyped (e.g., EnvA) RABV ΔG. This allows the tracing of the presynaptic partners of specific neurons. **(C–E)** RABV ΔG mediated expression of a number of genetic tools permit the dissection of structure/function of specific networks in a temporally and spatially controlled manner. **(C)** RABV ΔG mediated expression of recombinases (Cre/FLP) permits loss- or gain-of-function studies through conditional expression of specific genes in the infected network. **(D)** Population activity of specific neuronal networks can be monitored, for instance, by RABV ΔG directed expression of calcium indicators. **(E)** The activity of the starter cells, and their presynaptic network can be very specifically controlled by the expression of either allatostatin receptor or light-activated ion channels. Binding of allatostatin to its cognate receptor or activation of these channels by light of a specific wavelength leads to specific inhibition/activation of RABV ΔG infected neurons.

One way to achieve this is by taking advantage of an ever-increasing list of mice expressing cre-recombinase in specific neuronal cell types (e.g., reference Gong et al., 2003). TVA/RG is then selectively expressed in these cell types using a cre-dependent viral vector (Wall et al., 2010; Watabe-Uchida et al., 2012) (Figure 2A). This strategy was applied to map reciprocal synaptic connections between PKC-δ^−^ and PKC-δ^+^ interneurons in the central amygdala (Haubensak et al., 2010), and to perform brain-wide labeling of neurons providing direct input to dopaminergic neurons of the midbrain (Watabe-Uchida et al., 2012).

Alternatively, neuron types can be targeted based on their expression of cell surface receptors that recognize specific ligands. For example, a specific class of interneurons in the neocortex expresses the receptor ErbB4 that binds the soluble ligand neuregulin β 1. A recent study took advantage of this interaction to target ErbB4-positive neurons by pseudotyping RABV ΔG with an avian virus envelope protein (EnvB) that binds to a neuregulin β 1-EnvB receptor (TVB) bridge protein (Choi and Callaway, 2011). A lentivirus was pseudotyped in the same way to permit RG expression in the same source cell population (Choi and Callaway, 2011). Such approaches may also be of interest for studies of schizophrenia, which have been linked to a dysfunction in neuregulin signaling (Mei and Xiong, 2008). This approach could also provide a means for targeting RABV ΔG infection to particular cell populations in situations where genetic-based targeting approaches are not available, for example in primate systems (Choi and Callaway, 2011).

An alternative way to target a very specific source cell population is the use of a helper virus capable of propagating exclusively in dividing cells. Certain retroviruses, such as the Moloney murine leukemia virus (MMLV), are restricted in this manner and have classically been used to mark newly born neurons in the dentate gyrus of the hippocampus (reviewed in van Praag et al., 2002; Ming and Song, 2005). Stereotaxic injection of the MMLV expressing TVA/RG/fluorescent marker, followed by subsequent injection of RABV ΔG(EnvA) was thus used as a means of following the functional integration of newly born granule cells into existing neuronal circuits (Vivar et al., 2012).

All of the aforementioned examples necessitate two sequential stereotaxic injections into the same brain region [e.g., virus expressing TVA/RG, followed by RABV ΔG(EnvA)]. One can bypass the first step of this procedure by using RABV ΔG(EnvA) together with a bi-transgenic mouse line, in which TVA/RG is induced in a brain region- or cell-type specific manner under the control of a tet-regulatable promoter (Weible et al., 2010). This approach is suited to tracing long-range connections between nuclei that can be discretely targeted by cell-specific tetracycline transactivator expression. However, carefully designed controls are required to ensure that RG expression only occurs in the targeted source cell population and not in directly presynaptic neurons, as this would confound the interpretation of the tracing results (first-order vs. higher-order connectivity).

Mono-trans-synaptic tracing from defined source cell populations can also be achieved using a simpler targeting strategy which eliminates the need for the EnvA-TVA system, and instead takes advantage of the inherent qualities of certain retrogradely-transported viruses. Recent studies utilized this approach, by co-injecting RABV ΔG pseudotyped with its native G together with an RG-expressing retrogradely transported helper virus (Stepien et al., 2010; Yonehara et al., 2011). The source cell population was thus defined by their specific projection characteristics, rather than by their molecular identity. Injections were made into known target regions for the axons of certain neurons, thereby permitting the infection of axon terminals. With this approach, mono-trans-synaptic tracing enabled the identification of direct synaptic inputs to motor neurons targeting discrete muscle groups (Stepien et al., 2010), and to ON direction-selective retinal ganglion cells (Yonehara et al., 2011).

### Strategies to target individual neurons

The aforementioned strategies (sections “Targeting specific brain regions” and “Targeting a defined neuronal cell population”) may not permit the unambiguous identification of the presynaptic partners of a given neuron, because more than one source cell is typically targeted. Furthermore, in situations where a presynaptic partner of a source cell also expresses RG, an additional trans-synaptic crossing step may occur, resulting in the labeling of higher-order connections. One strategy to reduce this ambiguity is exemplified by Arenkiel et al. (2011). In this study the authors developed an elegant strategy to allow mono-trans-synaptic tracing of a very sparse and defined neuronal population. They transfected neuronal progenitor cells in the subventricular zone of the lateral ventricles in early postnatal mice with a targeting construct expressing TVA/RG. Adult-born granule cells derived from these progenitors migrate to the olfactory bulb and establish functional connections (Panzanelli et al., 2009). However, only a small number of migrated, functionally integrated daughter cells retained the ability to express TVA/RG, by virtue of rare integration events leading to stable expression of this targeting construct (Arenkiel et al., 2011). These source cells were then infected with RABV ΔG(EnvA) by stereotaxic injection into the olfactory bulb. The authors were thus able to trace microcircuits derived from sparsely distributed individual source granule cells.

The ability to target sparsely-distributed source cells may also be important for studies of long-range connections, where the topography or convergence of inputs needs to be examined and therefore the source cell number needs to be strictly limited. To achieve this, a complex strategy involving a conditional bi-transgenic mouse line and an AAV vector expressing TVA/RG under the control of a tetracycline-dependent promoter was employed (Miyamichi et al., 2011). RABV ΔG(EnvA) was subsequently injected into the same region as the AAV helper virus 14 days later. These tools not only permit tracing in a very sparse neuronal population, but also precise temporal control over TVA/RG expression.

Although mono-trans-synaptic tracing has most frequently been applied to populations of source cells, it also has great power for determining inputs into a single neuron *in vitro* and *in vivo* (Figure 2B). To target a single neuron, the plasmids encoding the tracing components (TVA/RG) and a fluorescent marker are first introduced together into this neuron. Two approaches have thus far been employed to achieve this *in vivo*—two-photon-guided electroporation and whole cell recording (Marshel et al., 2010; Rancz et al., 2011). Such *in vivo* approaches are painstaking, but permit the unambiguous identification of neurons presynaptic to a defined individual neuron. Furthermore, when combined with for instance whole-cell recordings and sensory stimulation, information regarding the anatomical receptive field of this neuron can be linked with its synaptic receptive field (Rancz et al., 2011).

## Recent advances in neuronal circuit analysis employing the RABV ΔG approach

### Mapping of long-range connections

One of the great advantages of RABV ΔG mediated mono-trans-synaptic tracing over other assays for synaptic connections [e.g., electron microscopy (EM) and paired electrophysiological recordings] is the ability to identify long-range synaptic connections. For example, RABV ΔG has been used to map direct long-range connections between mitral cells in the olfactory bulb and their postsynaptic targets in basal forebrain regions (Miyamichi et al., 2011). Such connections had previously been inferred from the presence of axons (e.g., reference Scott et al., 1980), but direct synaptic connectivity had not been proven. Similarly, Knobloch et al. (2012) employed the RABV ΔG technology as part of a complex toolkit to elucidate novel connections between oxytocin expressing neurons in the hypothalamus and neurons of the central amygdala. In this case, synaptic connections were strongly suggested by a variety of supporting evidence, obtained from optogenetic-based mapping and electron microscopy data. However, mono-trans-synaptic tracing was essential for confirming the presence of a functional synapse.

RABV ΔG based tracing approaches have also been exploited to map inputs into specific dopaminergic populations of the midbrain—in this case, on the scale of the whole brain (Watabe-Uchida et al., 2012). The authors were able to correct or refine previous findings obtained with conventional tracing or optogenetic approaches. By targeting genetically defined source cell populations they identified more specific subsets of presynaptically connected neurons. In addition, the authors showed that striatal neurons do in fact provide direct input to dopaminergic neurons of the ventral tegmental area or substantia nigra (Watabe-Uchida et al., 2012). Lastly, the exquisite labeling of the neuronal morphology afforded by RABV-driven eGFP expression aided the identification of the type of presynaptic neurons.

### Functional integration of postnatal-born neurons into a neural circuit

In addition to the validation of known connections, or the discovery of novel connections, a recent study examined the plasticity-induced remodeling of neural circuits (Arenkiel et al., 2011). In this study, the authors traced the connections formed by postnatal-born, newly migrated, differentiated granule cells following their integration into established circuits of the olfactory bulb. The authors further showed that odor stimulation resulted in increased input onto these neurons, as well as remodeling of their dendritic morphology. This study demonstrates the immense power of RABV ΔG based monosynaptic tracing for both screening of neural circuit changes as well as for fine morphological analysis.

A different study (Vivar et al., 2012) describes the time-dependent incorporation of adult-born granule cells of the dentate gyrus into existing neuronal circuits. To do this, the authors performed mono-trans-synaptic tracing at various time points after the initial labeling of adult-born daughter cells. They were then able to demonstrate quantitative changes in the type and number of the presynaptic neurons over time. In particular, they showed a transient period of input coming from mature granule cells, and that intra-hippocampal and cortical inputs increased with time. They also identified a novel “back-projection” from CA3 pyramidal cells. This study demonstrates the utility of RABV ΔG based mono-trans-synaptic tracing for the identification of novel connections, as well as for studying the remodeling of circuits.

### Three-dimensional topography of presynaptic neurons

With the use of RABV ΔG based trans-synaptic tracing, Stepien et al. (2010) were able to demonstrate that distinct populations of premotor neurons project to functionally-defined motor neuron pools, and that these populations extend over a large three-dimensional space along the spinal cord. In a related study the authors showed that premotor interneurons controlling antagonistic extensor-flexor muscles are segregated from one another along the medial lateral axis of the dorsal spinal cord (Tripodi et al., 2011). In an unrelated study Miyamichi et al. (2011) examined the topography of inputs from olfactory bulb into different olfactory cortex regions. In doing so, the authors were able to correlate topography of post/presynaptic neurons and divergence/convergence of connections with the presumed functionality of each type of olfactory processing center. These studies demonstrate that the topography of connections can yield additional information about the functionality of circuits.

### Novel RABV ΔG variants and their utility for neuronal circuit analysis

A range of RABV ΔG variants have recently been described. These variants include vectors expressing the *trans*-acting factors required for conditional expression of other transgenes (e.g., Cre, FLP recombinase; Figure 2C), a genetically encoded calcium sensor (Osakada et al., 2011; Kiritani et al., 2012) (Figure 2D) and molecules for the activation^51^ or silencing of circuits (Osakada et al., 2011) [e.g., allatostatin receptor, channelrhodopsin-2 (ChR2); Figure 2E]. Variants expressing additional fluorescent markers to permit a greater array of tracing possibilities are also described (Wickersham et al., 2007b; Osakada et al., 2011). These aforementioned tools can be combined with a variety of mouse models, for example those expressing Cre-or FLP-dependent reporters or conditional mutations permitting loss- or gain-of-function studies in defined neuronal circuits. A RABV ΔG vector expressing the light-activatable ChR2 has recently been used for the analysis of defined spino-cortical and cortico-striatal circuits (Apicella et al., 2012; Kiritani et al., 2012).

## Perspectives for the analysis of neural circuit structure/function

To date, our understanding of the wiring diagrams of neuronal circuits and the spatiotemporal dynamics of their electrical signals is rather limited. Critical steps toward this goal are the identification of the inputs of the circuit's diverse neuron types, and an understanding of their interaction within the circuit. The last years have seen numerous innovations in methods that rapidly advance the analysis of neural circuits. These include improvements in optical methods for stimulation and imaging, genetics, and computational methods. In the next paragraphs we will discuss how the RABV ΔG technology in concert with these methods can help shed light on some of the outstanding questions in this field.

### Tracing structure—from the nanoscale to the mesoscopic scale

RABV ΔG has outstanding properties for the labeling of the fine-scale neuronal morphology, due to its high-level expression of fluorescent proteins (Wickersham et al., 2007b). This feature is advantageous for visualization approaches where further signal amplification is not feasible. Such approaches include fluorescence imaging of entire mouse brains following a chemical clearing procedure (Dodt et al., 2007; Hama et al., 2011) or using serial two-photon tomography (STP tomography) (Ragan et al., 2012). Similarly, the high-intensity expression of morphological markers permits the dynamic observation of processes such as spine stability/turnover in living animals (for a recent review see Holtmaat and Svoboda, 2009), and likely satisfies the requirements for super-resolution imaging at nanoscale resolution (for review, see references Huang et al., 2009; Sigrist and Sabatini, 2012). Furthermore, RABV mediated expression of horseradish peroxidase may be a way to enhance the intensity of labeling required for ultrastructural techniques such as serial block-face scanning electron microscopy (Denk and Horstmann, 2004; Briggman et al., 2011; Li et al., 2011).

RABV ΔG pseudotyped with RG can be used as a retrograde tracer (Figure 1D) to infer connectivity between brain regions by labeling cells projecting to a specific target region (Wickersham et al., 2007a; Apicella et al., 2012; Kiritani et al., 2012). In combination with large-scale fluorescence imaging techniques (Dodt et al., 2007; Hama et al., 2011; Ragan et al., 2012) this approach allows systematic brain-wide mapping of neuronal circuits at the mesoscopic scale (Bohland et al., 2009) (see also Mouse Brain Architecture Project).

Thus, the RABV ΔG technology has the potential to aid visualization of neuronal morphology over spatial dimensions ranging from the nanoscale (electron microscopy/super-resolution light microscopy) to the entire mouse brain (light-sheet microscopy/STP tomography).

### Classifying neuronal cell types

One of the intractable problems with neuronal circuit analysis is the ability to define the neuronal cell types present within such a circuit. Cellular identity is based on an ever-more refined list of criteria such as electrical properties, patterns of gene expression and morphological features (Petilla Interneuron Nomenclature Group et al., 2008; Arenkiel and Ehlers, 2009). Ideally one would also link cell identity to the expression of a fluorescent marker to facilitate analysis of the properties of these cells within a circuit (Arenkiel and Ehlers, 2009; Lichtman and Denk, 2011; Steinmeyer and Yanik, 2012). For certain cell types this has been achieved with high specificity in transgenic mice (Gong et al., 2003) (reviewed in reference Lichtman and Denk, 2011). Pseudotyping of RABV ΔG permits the targeting of specific cellular populations, for example projection neurons or those expressing a certain surface protein (Mebatsion et al., 1997; Choi and Callaway, 2011). This enables the identification/classification of a neuron based on its morphology, projections, and in the case of mono-trans-synaptic tracing their connectivity properties. In the latter case, the morphological labeling of the presynaptic neurons aids their identification as well. RABV ΔG technology has been used, for example, to characterize neurons in the primate visual cortex (Nassi and Callaway, 2007; Nhan and Callaway, 2012) rodent neocortex (Larsen, 2008; Marshel et al., 2010; Rancz et al., 2011; Apicella et al., 2012; Kiritani et al., 2012) and rodent midbrain together with their presynaptic partners (Watabe-Uchida et al., 2012).

### Revealing synaptic connectivity

The construction of neural circuit connectomes requires comprehensive knowledge of the underlying synaptic connectivity. We are still lacking quantitative measurements regarding the numbers and sub-cellular organization of the different inputs received by a neuron. EM, paired electrophysiological recording, and optical simulation approaches have done much to advance our knowledge of synaptic connections between neurons. However, these approaches are either time-intensive (EM, paired recordings), currently limited to microcircuits (EM), or can only demonstrate the existence of synaptic connections, but not their precise sub-cellular location (paired recordings, optical stimulation methods).

**Figure 3:**
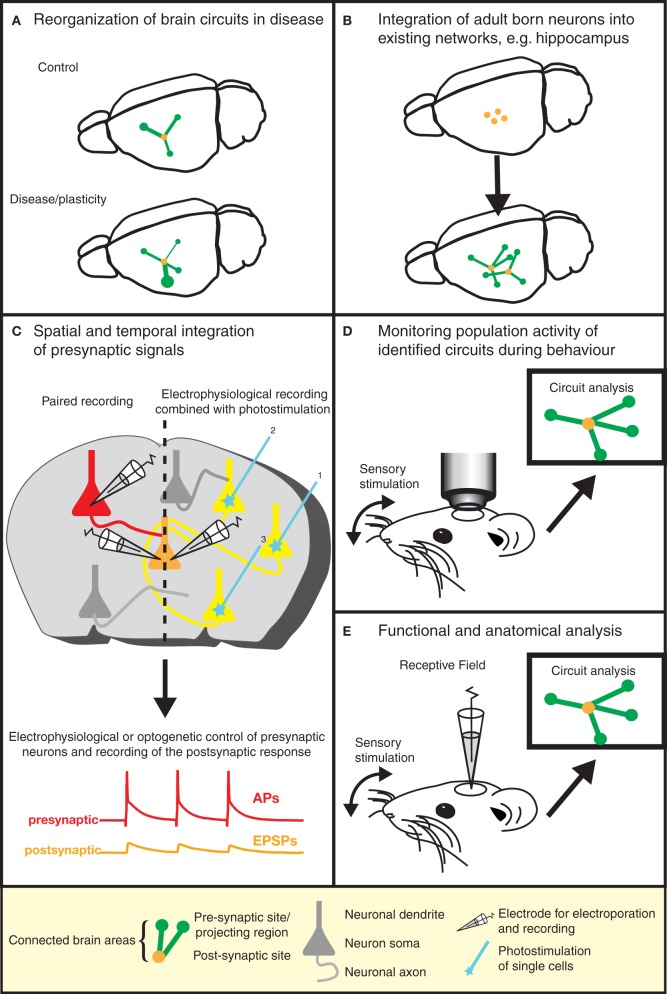
**Gaining insight into brain function by the dissection of neuronal circuits.** Recent advances in RABV ΔG technology provide invaluable tools for gaining a better understanding of the function and structure of neuronal circuits. **(A)** For instance, the re-organization of neuronal circuits resulting from plasticity or disease can be revealed by comparing the wiring diagram of experimental and control brains. **(B)** We can learn more about plasticity in the adult brain by studying the integration of adult-born neurons into existing neuronal networks. Similar approaches might be used to study circuit integration following stem cell therapy. **(C)** The analysis of the physiological properties of synaptic connections between defined neuron types (in addition to the anatomical wiring diagram) is greatly aided by this technique. Furthermore, the spatial and temporal integration of signals from a larger number of presynaptic neurons can be examined in postsynaptic neurons. **(D)** The population activity of a defined neuronal circuit can also be measured during active sensory stimulation or behavior and subsequently correlated with the anatomical input network of a defined starter population. **(E)** The receptive field properties of individual neurons can be combined with the analysis of the anatomical wiring diagram of their presynaptic network.

The elegance and power of the RABV ΔG mono-trans-synaptic tracing has now been amply demonstrated (as discussed above). Specifically, this technology provides information about the type, localization, and number of presynaptic neurons forming synaptic connections with particular target cell types. As such, it can be used for the validation of known neuronal synaptic connections (e.g., Stepien et al., 2010), but may also reveal hitherto unknown connections or a re-organization of wiring diagrams under physiological and pathophysiological conditions (Figure 3A). For example, the question of whether memories are stored in the connections formed by neurons (Seung, 2009) could be tested using this approach. Similarly, the integration of adult-born neurons (Arenkiel and Ehlers, 2009) into functional neuronal circuits can be examined (Figure 3B). A range of targeting approaches is now available (see above) to permit experimental design to be tailored to meet the requirements of a particular study. Lastly, in combination with techniques labeling synaptic contacts (GRASP/BLINC), mono-trans-synaptic tracing may be used to identify the sub-cellular location of synaptic inputs (Kim et al., 2012; Wickersham and Feinberg, 2012).

### Probing synaptic properties and synaptic integration

One important parameter defining neuronal circuits is the physiological properties of specific anatomical connections (i.e., release probability, strength, short-term dynamics etc.). So far, the most precise method to achieve this is the use of simultaneous electrophysiological recordings between synaptically connected neurons (e.g., reference Frick et al., 2008). Specific labeling of pre- and post-synaptic neurons with mono-trans-synaptic tracing greatly enhances the sampling efficiency of synaptic connections for a given postsynaptic neuron (Wickersham et al., 2007b) (Figure 3C). In addition, the integration of synaptic inputs from a number of presynaptic neurons within an individual neuron can be studied. As an alternative approach to simultaneous electrophysiological recordings, presynaptic partners could be stimulated optically following RABV ΔG driven expression of light-activated ion channels or heterologous receptor systems (Osakada et al., 2011) (Figures 2E and 3C).

### Combining physiology with architecture

Information processing in neural circuits depends on the spatiotemporal dynamics of the electrical signals within its neurons. It is therefore desirable to measure or manipulate the activity of these neurons during a particular behavioral task and to link the cellular activity with the circuit's structure (Figures 2D,E, and 3D). Electrophysiology is the most direct method to measure electrical activity, and provides a very high signal-to-noise ratio. Recently, it has become feasible to combine direct electrophysiological measurements of individual neurons *in vivo* with RABV ΔG mono-trans-synaptic tracing (Rancz et al., 2011) (Figures 2B and 3E).

**Figure 4:**
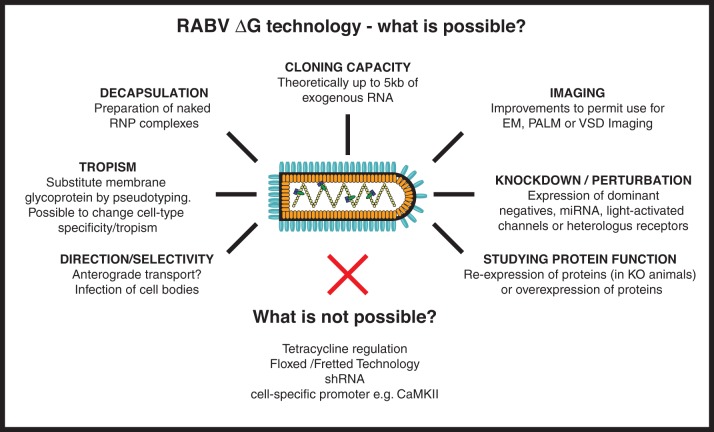
**RABV ΔG virus technology—what is possible?** Here we provide an overview of the numerous tools that could, in the future, be developed using RABV ΔG vectors. Such possibilities include the expression of genetically encoded tools to aid imaging/microscopy approaches and tools to examine loss-of-function (knockdown) or gain-of-function of a gene/protein of interest. Other possibilities involve retargeting using engineered envelope proteins or the possibility of transfection using naked ribonucleoprotein (RNP) complexes. Some approaches are not possible with RABV ΔG vectors including the integration of lox P sites, cell specific promoters or tet-regulatory sequences, or expression of shRNA. RNP, Ribonucleoprotein, core of the RABV; EM, Electron microscopy; PALM, Photo-activated localization microscopy; VSD, Voltage-sensitive dye; miRNA, micro RNA; shRNA, short hairpin RNA.

In other instances it is advantageous to use imaging approaches to monitor the cellular activity of identified populations of neurons during a specific task (Figure 3D). The advent of genetically-encoded indicators for calcium ions, membrane potential, and neurotransmitters (for a recent review, see reference Looger and Griesbeck, 2012) makes it possible to express such sensors of cellular activity using RABV ΔG technology (Osakada et al., 2011) (Figure 2D). For instance, the cellular resolution afforded by two-photon microscopy imaging of changes in calcium ions provide a proxy for action potentials in neuronal populations *in vivo* (recently reviewed in reference Grewe et al., 2010). Although measurements of changes in membrane potential provide a more direct readout of electrical activity, the lack of suitable genetically encoded voltage sensors has, so far, hampered *in vivo* measurements of neuronal activity with cellular resolution. A new class of voltage sensors may permit this notion to become a reality (Kralj et al., 2012; Looger and Griesbeck, 2012). These sensors can be readily incorporated into the RABV ΔG expression system for combined functional neural circuit studies. Further developments in the form of new variants (see Figure 4) are likely to increase the range of applications for which the unique properties of RABV ΔG vectors can be exploited for imaging of neuronal circuits in the intact animal.

Finally, gain- or loss-of-function experiments can also be implemented employing the RABV ΔG technology (Osakada et al., 2011). There is now a large selection of available light-activated ion channels and heterologous receptors to control neuronal excitability (for reviews see references Arenkiel and Ehlers, 2009; Fortin et al., 2011; Yizhar et al., 2011). For example, specific firing patterns can be triggered or neurons be silenced in targeted neuronal circuits (as defined by the RABV ΔG transfection strategy) within freely moving mice. This optical control of neuronal activity can then be integrated with measurements of the compatible readout, such as behavior, fluorescence measurements, electrical recordings, or fMRI signals (reviewed in reference Yizhar et al., 2011), and finally linked to the structural circuit information.

## Shortcomings of this approach and alternative monosynaptic viral tracers

### Undersampling of presynaptic neurons

Although immensely powerful, RABV ΔG based tracing is, like any technology, affected by particular shortcomings. Certainly the most perplexing issue is the undersampling of labeled presynaptic neurons. This has been most clearly demonstrated by targeting single pyramidal neurons in the primary visual cortex *in vivo* (Marshel et al., 2010; Rancz et al., 2011). Mono-trans-synaptic tracing from individual layer 2/3 pyramidal neurons resulted in the labeling of fewer than 100 presynaptic neurons (Marshel et al., 2010), while up to ~700 presynaptic neurons were labeled by targeting individual layer 5 pyramidal neurons (Rancz et al., 2011). Furthermore, mono-trans-synaptic tracing from a single starter cell in layer 5 of the S2 somatosensory cortex resulted in the tracing of 249 presynaptic cells (Miyamichi et al., 2011). These differences may be partially due to cell type and/or other factors such as plasmid delivery or regulation of transgene expression. Nonetheless, the number of labeled presynaptic neurons was substantially lower than that expected from calculations based on dendritic spine numbers and the average number of synapses formed between connected pairs of neocortical pyramidal neurons (e.g., references Larkman, 1991; Defelipe and Farinas, 1992; Markram et al., 1997; Feldmeyer et al., 2002). One possible explanation for this undersampling is the level of expression of RG which may fail to meet the stoichiometry required for the efficient assembly and trans-synaptic crossing of RABV particles *in vivo* (Marshel et al., 2010). It remains to be shown whether certain properties of the synaptic contacts (strength, activity, or molecular signature of presynaptic neuron) could influence the efficacy of synaptic crossing. A recently developed single-cell targeting technique using a recombinant polysynaptic RABV (Nguyen et al., 2012) should help shed some light on this issue, as should a more detailed analysis of the molecular characteristics of the synapses that are efficiently crossed.

### Neuroinvasiveness

At least one study has pointed to a limited time window for experimental infection by RABV ΔG following intramuscular injection. Stepien et al. (2010) reported that motor neurons are refractory to infection by the peripheral route after approximately postnatal day 10. This limitation most likely stems from the fact that the RABV ΔG mono-trans-synaptic tracing approach employs a recombinant virus derived from the Street Alabama Dufferin (SAD) B19 strain of RABV. This strain is highly attenuated for infection by the peripheral route and is only neuroinvasive following peripheral injection in young mice (Finke and Conzelmann, 2005; Rasalingam et al., 2005; Dietzschold et al., 2008). This may perhaps lead to the misconception that RABV ΔG technology can only be applied over a limited postnatal time window. For direct injections into the CNS, however, there is no such impediment. Efficient tracing has been observed in a variety of brain areas in both rats and mice of 1–6 months of age (Haubensak et al., 2010; Arenkiel et al., 2011; Rancz et al., 2011; Knobloch et al., 2012; Watabe-Uchida et al., 2012).

### Time window for tracing

Features that make RABV extremely efficient both as pathogen and as viral tracer are also associated with its reduced cytotoxicity (Dietzschold et al., 2008; Lafon, 2011; Rieder and Conzelmann, 2011; Ugolini, 2011). Deleting RG is suggested to further decrease its cytotoxicity (Wickersham et al., 2010). Nevertheless, there is a finite time window for RABV ΔG mediated tracing experiments. RABV ΔG infected cortico-thalamic neurons maintain normal electrophysiological parameters up to 12 days following infection (Wickersham et al., 2007b). However, morphological defects or cell death were observed from 14–16 days post-infection (Wickersham et al., 2007b; Arenkiel et al., 2011). Osakada et al. (2011) suggest a 5–11 days (post-infection) time window for viable physiological experiments, which would also take into account any increased toxicity related to the nature of the virally expressed protein. Altogether, these data suggest a sufficiently broad time-window for RABV ΔG based tracing experiments. These findings may be contrasted with the significant perturbations in neuronal physiology that were reported to occur as early as 72 h post-infection with a GCaMP2-expressing PRV strain derived from the highly attenuated PRV-Bartha (Granstedt et al., 2009).

### Alternative virus-based mono-trans-synaptic tracing techniques

In spite of the many advantages of RABV ΔG, this technology is currently restricted to mapping *input* to a neuronal population. An alternative approach, permitting anterograde trans-synaptic tracing from a defined source cell population was recently described (Lo and Anderson, 2011). This approach employs a modified, thymidine kinase-deleted herpes simplex virus 1 (HSV-1) derived from strain 129 and capable of propagating in a selective manner in cre-expressing starter cells. This virus was tested in a variety of cre-expressing transgenic mouse lines and resulted in reliable anterograde trans-synaptic mapping of defined neuronal circuits. Notably, this system can be readily combined with the large number of existing cre-/BAC-cre transgenic mouse lines and appears to be specific with respect to trans-synaptic transmission. Unfortunately, this approach is limited to the extent that it is not monosynaptic. In addition, it suffers from the cytotoxicity inherent to HSV-1 viruses and may also have the disadvantage of infecting axon terminals at the site of injection (Lo and Anderson, 2011). A cell-targetable, anterograde monosynaptic tracer derived from a glycoprotein-deleted vesicular stomatitis virus has recently been described (Beier et al., 2011). However, it is uncertain whether these findings can be reproduced *in vivo* (see Beier et al., 2012).

## Concluding remarks

Recent developments in the field of RABV ΔG technology convincingly establish the importance and power of this tool for neural circuit mapping. Combined with other imaging, physiological, and behavioral approaches, it promises to open new avenues of research permitting a greater understanding of the mammalian connectome and its function. RABV ΔG is applicable to a range of experimental paradigms and permits not only the study of circuit structure, but also the ability to manipulate or monitor its function.

RABV ΔG is invaluable as a viral tracer. In addition, it has considerable potential as a neuronal expression vector. In contrast to classical polysynaptic viral tracers, the current technology permits a greater degree of control over initially infected source cells, by virtue of pseudotyping technology and other genetic targeting approaches. RABV ΔG technology allows a comprehensive reconstruction of the input map of defined neurons as well as physiological monitoring of defined neuronal populations. This tool shows great promise for improving our understanding of neuronal circuit structure/function and its plastic modification during physiological processes or in disease. Future strategies for neuronal circuit analysis will involve a close alliance of genetics, circuit tracing, light- or heterologous receptor-mediated control, high-resolution imaging, and electrophysiology, and RABV ΔG will be an essential part of this tool box.

### Conflict of interest statement

The authors declare that the research was conducted in the absence of any commercial or financial relationships that could be construed as a potential conflict of interest.

## Glossary

*Anterograde tracing*: Tracing of projection neurons by transport from the cell bodies to the axon terminals.
*Connectome*: Here referred to as a complete map of all synaptic connections of the brain or a neural circuit.
*Mono-trans-synaptic tracing or monosynaptic trans-synaptic tracing*: The process of tracing connections between source cells and first-order synaptically connected partners.
*Neurotropic virus*: Virus preferentially infecting neurons.
*Polysynaptic tracer*: This term is often used quite loosely to describe tracers that spread from one neuron to another (trans-neuronal tracer), but not always exclusively between synaptically connected cells. In contrast to mono-trans-synaptic tracers, tracing can occur between chains of connected neurons.
*Pseudotyping*: The process of replacing the native surface glycoprotein of a virus with that of another virus to alter viral tropism.
*Retrograde tracing*: Tracing of projection neurons by transport from the axon terminals to the cell bodies.
*Reverse genetics*: In the context of virology the term reverse genetics implies the reconstitution of live infectious virus particles using cDNAs, transfected into heterologous cells, as starting material.
*Source cells*: Initially infected postsynaptic neuron/s that allows retrograde RABV ΔG spread upon RG transcomplementation, thereby revealing directly presynaptic partners.
*Trans-neuronal tracer*: A tracer that passes from one neuron to another, for which synapse specificity has not been adequately identified.
*Trans-synaptic tracer*: A tracer that spreads exclusively between neurons that are connected by synaptic contacts.
*Tropism*: Preferential or specific infection of, and replication within, a certain cell-type.
